# Nine Whole-Genome Assemblies of *Yersinia pestis* subsp. *microtus* bv. Altaica Strains Isolated from the Altai Mountain Natural Plague Focus (No. 36) in Russia

**DOI:** 10.1128/genomeA.01440-17

**Published:** 2018-01-18

**Authors:** Angelina A. Kislichkina, Alexandr G. Bogun, Lidiya A. Kadnikova, Nadezhda V. Maiskaya, Viktor I. Solomentsev, Svetlana V. Dentovskaya, Sergey V. Balakhonov, Andrey P. Anisimov

**Affiliations:** a State Research Center for Applied Microbiology and Biotechnology, Obolensk, Russian Federation; b Irkutsk Antiplague Research Institute of Siberia and the Far East, Irkutsk, Russian Federation

## Abstract

We report here the draft genome sequences of nine *Yersinia pestis* subsp. *microtus* bv. Altaica strains isolated from the Altai Mountain plague focus (no. 36), which represent the 0.PE4 phylogroup circulating in populations of Mongolian pika (*Ochotona pallasi*).

## GENOME ANNOUNCEMENT

The plague is a zoonotic infection caused by the Gram-negative bacterium *Yersinia pestis*, which is occasionally transmitted to humans from infected rodents through the bites of infected fleas. The majority of the natural plague foci are not geographically connected, resulting in considerable ecological differences needed for *Y. pestis* to survive and be transmitted in these different environments. Adaptation to dissimilar living conditions led to the formation of considerable diversity in genotypes and phenotypes among *Y. pestis* isolates from different natural foci.

The Altai Mountain natural plague focus (no. 36) is the Russian part of the Sailugem natural plague focus located in the northwest of Mongolia (1). The main components of this natural focus are populations of *Y. pestis* 0.PE4 phylogroup strains belonging to *Y. pestis* subsp. *microtus* and characterized by high virulence to their main host (*Ochotona pallasi*) and laboratory mice but almost always avirulence or weak virulence for guinea pigs and humans (2). Although this plague focus is characterized by permanent epizootic activity, its epidemic potential is low, and there were no registered cases of the disease in people (3).

DNA samples were extracted using conventional SDS lysis and phenol-chloroform extraction methods.

Whole-genome sequencing was performed using an Illumina MiSeq instrument according to the manufacturer’s instruction. DNA libraries were prepared using the Nextera DNA laboratory preparation kit. A MiSeq reagent kit v3 was used for sequencing. For each genome, reads were assembled *de novo* using SPAdes v.3.8.1 (http://cab.spbu.ru/software/spades/). Finally, we obtained from 167 to 211 contigs for each genome (Table 1). The genome sizes ranged from 4.61 to 4.65 Mb. Each genome contains 4,255 to 4,511 genes. All strains except one have three plasmids (pMT, pCD, and pPCP). Strain I-3442 does not carry plasmid pPCP, but the other two plasmids are present.

**TABLE 1:** Strain-identifying information and basic statistics on assemblies and annotations

Strain name	Alternative strain name	SRA accession no. (raw data)	GenBank accession no.	Size (bp)	No. of contigs	No. of genes		Plasmid^a^		
						Total	Coding	pMT/pFra	pCD/pYV	pPCP/pPst
I-3442	SCPM-O-DNA-01	SRR4017182	NHYH00000000	4,641,984	181	4,449	4,205	+	+	−
I-3443	SCPM-O-DNA-02	SRR4017184	MIED00000000	4,646,108	167	4,255	4,054	+	+	+
I-3446	SCPM-O-DNA-03	SRR4017191	NHYI00000000	4,651,412	211	4,477	4,232	+	+	+
I-3447	SCPM-O-DNA-04	SRR4017192	MIEE00000000	4,646,075	167	4,268	4,053	+	+	+
I-3515	SCPM-O-DNA-05	SRR4017193	NHYJ00000000	4,650,263	187	4,465	4,225	+	+	+
I-3516	SCPM-O-DNA-06	SRR4017195	NHMW00000000	4,647,570	172	4,487	4,252	+	+	+
I-3517	SCPM-O-DNA-07	SRR4017197	NHMX00000000	4,612,130	182	4,469	4,241	+	+	+
I-3518	SCPM-O-DNA-08	SRR4017214	NHMY00000000	4,652,539	198	4,511	4,270	+	+	+
I-3519	SCPM-O-DNA-09	SRR4017215	NHMZ00000000	4,650,126	179	4,475	4,251	+	+	+

a −, not present; +, present.

A detailed report of a full comparative genomic analysis will be included in a future publication.

### Accession number(s).

The GenBank accession numbers for these nine genome sequences are listed in Table 1.
